# Editorial: Extracellular vesicles as modulators of cancer cell adaptive responses linked to therapy resistance

**DOI:** 10.3389/fonc.2022.1101103

**Published:** 2022-11-29

**Authors:** Antonio Giordano, Nadia Rucci, Stefano Falone

**Affiliations:** ^1^ Sbarro Institute for Cancer Research and Molecular Medicine, Center for Biotechnology, College of Science and Technology, Temple University, Philadelphia, PA, United States; ^2^ Department of Medical Biotechnologies, University of Siena, Siena, Italy; ^3^ Department of Biotechnological and Applied Clinical Sciences, University of L’Aquila, L’Aquila, Italy; ^4^ Department of Life, Health and Environmental Sciences, University of L’Aquila, L’Aquila, Italy

**Keywords:** cancer resistance, molecular cargoes, tumor microenvironment, cellular adaptive response, redox homeostasis, inflammation, extracellular vesicles (EVs)

Cancer still ranks as the leading cause of death, with approximately 20 million new cases per year worldwide (1). The main obstacle to cancer eradication is that anticancer approaches are often hindered by innate or acquired resistance to treatments (2). Cancer cells escape toxicity of therapeutics *via* genetic heterogeneity, enhanced pro-survival signals, metabolic reprogramming and improved detoxification and antioxidant scavenging, among other mechanisms (3–5). The crosstalk between tumor cells and the surrounding tumor microenvironment (TME) through the extracellular vesicle (EV)-based communication system plays a major role in influencing the behavior and phenotype of cancer cells through a wide array of molecular cargoes, such as proteins, nucleic acids, lipids, and metabolites (6, 7).

Unfortunately, a comprehensive view of the molecular mechanisms through which EVs affect resistance to anticancer treatments is yet to be depicted. Some evidence suggests that key roles might be played by regulatory RNAs (namely, lncRNAs and miRNAs) and drug efflux pumps (8, 9), metabolism reprogramming in cancer cells and in the TME (10), changes in mitochondrial function, bioenergetics, reactive oxygen species production and disposal, as well as in genomic stability and epigenetic control of gene expression (11–13).

The aim of this Research Topic was to collect contributions focused on how EVs affect molecular phenotype and behavior of cancer cells, in terms of their response to anticancer interventions.

Pompili et al. summarized the current state of knowledge on the most important cellular pathways involved in the cytoprotective effects of EVs in cancer cells, which can gain resistance to chemotherapy *via* EV-dependent extrusion of therapeutics, or even through the uptake of diverse molecular cargoes, including ABC transporter proteins, inhibitors of apoptosis, phase II detoxification enzymes, proliferation enhancers and non-coding RNAs. Similarly, Palazzolo et al. reviewed how EV molecular cargoes can change the response profile of cancer cells to chemotherapeutics, for example by inducing epithelial-mesenchymal transition (EMT) and cancer stem cell (CSC) phenotypes, by stimulating the expression of ATP-dependent efflux pumps, such as P-gp, or even impairing caspase 3-dependent apoptosis. Pompili et al. and Palazzolo et al. also discussed how natural or modified EVs may serve as drug delivery systems, and how the EV-dependent cell-to-cell communication may be targeted to reduce chemoresistance in cancer.

Studies provided evidence for an EV-mediated cell-to-cell transmission of drug-resistance traits in malignancies (14–16). The ability of EVs to transfer resistance to recipient cells was investigated by Lombardi et al., who observed that (TMZ)-sensitive glioblastoma multiforme cells became less responsive to TMZ after internalization of cyclooxygenase-2-containing EVs derived from TMZ-resistant cancer cells.

The involvement of the redox milieu in the EV-dependent modification of cancer cell behavior was investigated by some of us. Ponzetti et al. showed that osteoblast-derived EVs (OB-EVs) reduced osteosarcoma cells’ aggressiveness and viability by impairing the redox balance of glutathione, a critical endogenous antioxidant molecule with key functions in detoxification and reactive oxygen species (ROS) scavenging within cells (17). Interestingly, OB-EVs did not alter the energy-related metabolic balance or mitochondrial dynamics.

NF-κB plays a major role in the execution of redox cellular responses. As reviewed by Di Vito Nolfi et al., NF-κB, whose expression governs key pro-survival pathways, is positively regulated by the EV-dependent release of specific tumor-promoting factors in the TME. A reciprocal regulation exists between EVs and NF-κB signaling, with NF-κB being directly involved in EVs trafficking and EVs-mediated chemoresistance, along with EVs playing a role in the activation of NF-κB. The authors discussed also how other proteins, molecules, molecular mechanisms and pathways possibly play a role in chemoresistance. The EV-mediated intercellular communication contributes to pathway activation, immune escape, and drug resistance Di Vito Nolfi et al. Beyond its important role in the redox response, NF-κB regulates an array of genes involved in immune and inflammatory responses (18). Mezzasoma et al. summarized how EVs and the pro-inflammatory TME could lead to cancer drug resistance, for example by modulating the activity of the NLRP3-dependent cascade, thus altering the inflammasome activation in cancerous recipient cells, as well as stimulating immune-escape or immune-stimulation, depending on the nature of the EV-releasing and -receiving cells. The authors also discussed how potential inhibitors of the inflammasome machinery could be effectively exploited to develop new anti-cancer strategies. Simón et al. also discussed how the proinflammatory TME elicits pro-survival effects through EV release. These authors summarized the role of hypoxia and chemotherapy in promoting release of EVs. Moreover, they described how macrophages and adipocytes, main contributors to pro-inflammatory disorders, can also induce release of EVs eventually leading to increased chemoresistance. Finally, the authors also provided an interesting picture of the pro-survival molecular pathways activated by CSC- and cancer associated fibroblast (CAF)-derived EVs. CAFs promote cancer progression by facilitating metastasization, angiogenesis, immunosuppression and drug resistance (19). In this context, Giusti et al. clearly demonstrated that tumor-derived EVs activate fibroblasts into a CAF-like phenotype, supporting their proliferation, motility, invasiveness and enzyme expression.

Increasing evidence underlines a crucial role for EVs within the TME as one of the main determinant for the immune function of neutrophils in malignancies (20). Zippoli et al. reviewed how tumor-derived EVs promote the differentiation of a pro-tumoral immune-suppressive sub-population of tumor associated neutrophils (TANs) and suppress T cell-mediated immunity by increasing the expression of programmed death-ligand 1 (PD-L1) in neutrophils. Interestingly, the authors reviewed also literature that suggests that neutrophil-derived EVs may serve as predictors of cancer outcome.

Lu et al. experimentally demonstrated that exosomes (EXOs) from dendritic cells infected with *Toxoplasma gondii* inhibited tumor growth in a mouse model of colorectal cancer (CRC), thus providing insights of how parasite-based anticancer strategies may achieve interesting results. Further research should identify the specific components of the exosomes involved in this effect.

The regulatory RNAs shuttled by EXOs may be involved in modulating the response to anticancer drugs (21). Wu et al. described the role of circRNAs shuttled by EVs as either suppressors or promoters of resistance to radiation in various cancer models. Accordingly, circRNAs could serve as novel clinical radiosensitizers, and as biomarkers to predict the effect of radiotherapy on tumors, thus providing a basis for targeted precision treatment in the future. In addition to the direct effect of radiation on irradiated cells, the authors also observed a process known as the radiation-induced bystander effect (RIBE), in which non-irradiated cells are also indirectly affected by radiation. RIBE appears to play a major role in determining the success of cancer radiotherapy. Further research is needed to identify if circRNAs can also induce RIBE through EXOs. As discussed in the mini-review from Zelli et al., exosomal miRNAs are highly biocompatible, scarcely immunogenic, and have the ability to cross the blood-brain barrier, thus representing potential therapeutic delivery agents to suppress or prevent further tumor progression.

EVs cargo also include lipids, such as cholesterol, ceramide, sphingomyelin, and phosphatidylserine. Interestingly, in the original article from Chen et al. atorvastatin was found to reduce the release of EVs and their lipid content in ovarian adenocarcinoma cells, while promoting the release of cholesterol-enriched EVs. These effects were linked to reduced cell proliferation, migration, invasion, and to an increase in chemosensitivity to paclitaxel.

Acquired resistance to drugs is a major cause for hepatocellular carcinoma (HCC) being a highly relapsing disease and a leading cause of cancer mortality (22). Wang et al. reviewed how specific HCC-derived cargoes promote the conversion of hepatic stellate cells to CAFs, induce a pro-angiogenic effect and reduce endothelial integrity, eventually promoting tumor invasion. In addition, the authors discussed how specific EVs-associated miRNAs could be used as valuable biomarkers for HCC diagnosis.

This guest editorial board hopes that the contributions here collected offer innovative and interesting mechanistic insights on the decisive role of EVs as key regulators of critical aspects of cancer cell phenotype and behavior, in terms of their capacity to stimulate the cellular stress response upon treatment, as well as in terms of their ability to enable cancer cells to escape death upon exposure to antitumor agents.

We wish to thank all the Authors for sharing novel findings and interesting views of the current state of understanding on this Research Topic. We also greatly appreciated the valuable support given by the independent experts during the peer-review of all the submitted manuscripts.

## Author contributions

AG: Conceptualization, Writing - Original Draft, Writing - Review & Editing; NR: Conceptualization, Writing - Original Draft, Writing - Review & Editing; SF: Conceptualization, Writing - Original Draft, Writing - Review & Editing. All authors contributed to the article and approved the submitted version.

## Acknowledgments

We wish to thank all the Authors for sharing novel findings and interesting views of the current state of understanding on this topic. We also greatly appreciated the valuable support given by the independent experts during the peer-review of all the submitted manuscripts.

## Conflict of interest

The authors declare that the research was conducted in the absence of any commercial or financial relationships that could be construed as a potential conflict of interest.

## Publisher’s note

All claims expressed in this article are solely those of the authors and do not necessarily represent those of their affiliated organizations, or those of the publisher, the editors and the reviewers. Any product that may be evaluated in this article, or claim that may be made by its manufacturer, is not guaranteed or endorsed by the publisher.

## References

[1] Sung H Ferlay J Siegel RL Laversanne M Soerjomataram I Jemal A. Global cancer statistics 2020: GLOBOCAN estimates of incidence and mortality worldwide for 36 cancers in 185 countries. CA Cancer J Clin (2021) 71(3):209–49. doi: 10.3322/caac.21660 33538338

[2] Wang X Zhang H Chen X. Drug resistance and combating drug resistance in cancer. Cancer Drug Resist (2019) 2(2):141–60. doi: 10.20517/cdr.2019.10 PMC8315569 34322663

[3] Fiorillo M Ózsvári B Sotgia F Lisanti MP. High ATP production fuels cancer drug resistance and metastasis: Implications for mitochondrial ATP depletion therapy. Front Oncol (2021) 11:740720. doi: 10.3389/fonc.2021.740720 34722292 PMC8554334

[4] Bukowski K Kciuk M Kontek R. Mechanisms of multidrug resistance in cancer chemotherapy. Int J Mol Sci (2020) 21:3233. doi: 10.3390/ijms21093233 32370233 PMC7247559

[5] Falone S Lisanti MP Domenicotti C. Oxidative stress and reprogramming of mitochondrial function and dynamics as targets to modulate cancer cell behavior and chemoresistance. Oxid Med Cell Longev (2019) 2019:4647807. doi: 10.1155/2019/4647807 31915507 PMC6930714

[6] Fridman ES Ginini L Gil Z. The role of extracellular vesicles in metabolic reprogramming of the tumor microenvironment. Cells (2022) 11:1433. doi: 10.3390/cells11091433 35563739 PMC9104192

[7] Bebelman MP Smit MJ Pegtel DM Baglio SR. Biogenesis and function of extracellular vesicles in cancer. Pharmacol Ther (2018) 188:1–11. doi: 10.1016/j.pharmthera.2018.02.013 29476772

[8] Guo C Liu J Zhou Q Song J Zhang Z Li Z. Exosomal noncoding RNAs and tumor drug resistance. Cancer Res (2020) 80:4307–13. doi: 10.1158/0008-5472.CAN-20-0032 32641408

[9] Xavier CPR Belisario DC Rebelo R Assaraf YG Giovannetti E Kopecka J. The role of extracellular vesicles in the transfer of drug resistance competences to cancer cells. Drug Resist Update (2022) 62:100833. doi: 10.1016/j.drup.2022.100833 35429792

[10] Yang E Wang X Gong Z Yu M Wu H Zhang D. Exosome-mediated metabolic reprogramming: the emerging role in tumor microenvironment remodeling and its influence on cancer progression. Signal Transduct Target Ther (2020) 5:242. doi: 10.1038/s41392-020-00359-5 33077737 PMC7572387

[11] Thakur A Johnson A Jacobs E Zhang K Chen J Wei Z. Energy sources for exosome communication in a cancer microenvironment. Cancers (2022) 14:1698. doi: 10.3390/cancers14071698 35406470 PMC8996881

[12] Chiaradia E Tancini B Emiliani C Delo F Pellegrino RM Tognoloni A. Extracellular vesicles under oxidative stress conditions: Biological properties and physiological roles. Cells (2021) 10:1763. doi: 10.3390/cells10071763 34359933 PMC8306565

[13] Di Liegro CM Schiera G Di Liegro I. Extracellular vesicle-associated RNA as a carrier of epigenetic information. Genes (2017) 8:240. doi: 10.3390/genes8100240 28937658 PMC5664090

[14] Corcoran C Rani S O’Brien K O’Neill A Prencipe M Sheikh R. Docetaxel-resistance in prostate cancer: evaluating associated phenotypic changes and potential for resistance transfer *via* exosomes. PloS One (2012) 7:e50999. doi: 10.1371/journal.pone.0050999 23251413 PMC3519481

[15] Ifergan I Scheffer GL Assaraf YG. Novel extracellular vesicles mediate an ABCG2-dependent anticancer drug sequestration and resistance. Cancer Res (2005) 65:10952–8. doi: 10.1158/0008-5472.CAN-05-2021 16322243

[16] Choi D-Y You S Jung JH Lee JC Rho JK Lee KY. Extracellular vesicles shed from gefitinib-resistant nonsmall cell lung cancer regulate the tumor microenvironment. Proteomics (2014) 14:1845–56. doi: 10.1002/pmic.201400008 24946052

[17] Sies H Berndt C Jones DP. Oxidative stress. Annu Rev Biochem (2017) 86:715–48. doi: 10.1146/annurev-biochem-061516-045037 28441057

[18] Oeckinghaus A Ghosh S. The NF- b family of transcription factors and its regulation. Cold Spring Harb Perspect Biol (2009) 1:a000034–a000034. doi: 10.1101/cshperspect.a000034 20066092 PMC2773619

[19] Eskandari-Malayeri F Rezaei M. Immune checkpoint inhibitors as mediators for immunosuppression by cancer-associated fibroblasts: A comprehensive review. Front Immunol (2022) 13:996145. doi: 10.3389/fimmu.2022.996145 36275750 PMC9581325

[20] Yang P Peng Y Feng Y Xu Z Feng P Cao J. Immune cell-derived extracellular vesicles - new strategies in cancer immunotherapy. Front Immunol (2021) 12:771551. doi: 10.3389/fimmu.2021.771551 34956197 PMC8694098

[21] Lou G Song X Yang F Wu S Wang J Chen Z. Exosomes derived from miR-122-modified adipose tissue-derived MSCs increase chemosensitivity of hepatocellular carcinoma. J Hematol Oncol (2015) 8:122. doi: 10.1186/s13045-015-0220-7 26514126 PMC4627430

[22] Lohitesh K Chowdhury R Mukherjee S. Resistance a major hindrance to chemotherapy in hepatocellular carcinoma: an insight. Cancer Cell Int (2018) 18:44. doi: 10.1186/s12935-018-0538-7 29568237 PMC5859782

